# Climatic warming and the future of bison as grazers

**DOI:** 10.1038/srep16738

**Published:** 2015-11-16

**Authors:** Joseph M. Craine, E. Gene Towne, Mary Miller, Noah Fierer

**Affiliations:** 1 Jonah Ventures, Manhattan KS 66502, USA; 2 Kansas State University, Manhattan KS 66502, USA; 3 The Nature Conservancy, Leola, SD 57456, USA; 4 Department of Ecology and Evolutionary Biology, University of Colorado, Boulder, CO 80309, USA; 5 Cooperative Institute for Research in Environmental Science, University of Colorado, Boulder, CO 80309.

## Abstract

Climatic warming is likely to exacerbate nutritional stress and reduce weight gain in large mammalian herbivores by reducing plant nutritional quality. Yet accurate predictions of the effects of climatic warming on herbivores are limited by a poor understanding of how herbivore diet varies along climate gradients. We utilized DNA metabarcoding to reconstruct seasonal variation in the diet of North American bison (*Bison bison*) in two grasslands that differ in mean annual temperature by 6 °C. Here, we show that associated with greater nutritional stress in warmer climates, bison consistently consumed fewer graminoids and more shrubs and forbs, i.e. eudicots. Bison in the warmer grassland consumed a lower proportion of C_3_ grass, but not a greater proportion of C_4_ grass. Instead, bison diet in the warmer grassland had a greater proportion of N_2_-fixing eudicots, regularly comprising >60% of their protein intake in spring and fall. Although bison have been considered strict grazers, as climatic warming reduces grass protein concentrations, bison may have to attempt to compensate by grazing less and browsing more. Promotion of high-protein, palatable eudicots or increasing the protein concentrations of grasses will be critical to minimizing warming-imposed nutritional stress for bison and perhaps other large mammalian herbivores.

Climatic warming has the potential to reduce the growth and reproduction of large mammalian terrestrial herbivores by not only reducing the nutritional quality of plant species, but also decreasing the relative abundance of nutritionally critical species^1^,^2^,^3^. For North American plains bison (*Bison bison*), a keystone species in North American grasslands^4^, climatic warming is likely to increase protein stress^1^, reducing bison growth and reproduction^2^. However, bison diet remains poorly resolved, which limits our ability to mitigate climatic warming by better managing dietarily critical plant species.

Plains bison are considered to be strict grazers^5^,^6^,^7^,^8^,^9^, which implies that they primarily consume grasses (Poaceae) and other monocots, e.g. sedges (*Carex*), as opposed to browsing on forbs, shrubs, or trees, i.e. herbaceous and woody eudicots^10^. Being strict grazers would suggest that climatic warming may reduce bison performance by altering the productivity and nutritional quality of different grass species. Yet, many of the previous diet reconstructions that led to considering bison as grazers were based on microhistological analyses, which likely overemphasized the contribution of grasses and underemphasized the amount of eudicot species in their diet^11^,^12^. Some studies have suggested that bison utilize eudicot species to some degree^13^,^14^,^15^,^16^, although the extent and regularity of eudicot consumption are unknown. If eudicot species constitute a critical component of bison diet, then managers will need to take into account the relative abundance of eudicot species on the landscape and their nutritional quality when considering mitigation strategies.

To better understand geographic patterns of bison dietary composition, we collected fresh bison fecal material at biweekly to monthly intervals in two North American humid grasslands (Kansas and South Dakota). The mean annual temperature of the Kansas grassland is 6 °C greater than the South Dakota grassland, which is slightly higher than the upper limit of projected temperature change for the region by 2100^17^. Bison at the Kansas site have lower body mass than bison at the South Dakota site (e.g. 3.5 y-old male bison in Kansas are 120 kg lighter on average than in South Dakota), which is likely due to higher temperatures lowering dietary quality^2^. Collected fecal material was pooled by sex and sampling period and then analyzed for plant DNA via high-throughput sequencing of a chloroplast intron (*trnL*)^18^,^19^, allowing for quantitative reconstruction of the taxonomic composition of diets. Sequence data were clustered into operational taxonomic units (OTUs) and assigned to taxa by comparing with a locally generated sequence library and a public sequence database.

DNA metabarcoding revealed that Kansas bison consumed a diverse diet of graminoids and eudicots. Eudicots represented a majority of the *trnL* reads recovered. For the Kansas bison across the entire three years, 39% of the sequences were from graminoids: 8 ± 1% (s.e.) were graminoids with the C_3_ photosynthetic pathway, 31 ± 2% were grasses with the C_4_ photosynthetic pathway. In contrast, 61% of the sequences were from eudicots: 18 ± 2% were from non-N_2_-fixing forbs and 43 ± 3% were from N_2_-fixing species. The temporal patterns of the plant functional groups in Kansas bison revealed that C_3_ graminoid consumption was highest during the winter (~20% from Nov 1 – Apr 1 vs. 3% for the rest of the year; Fig. 1). Consumption of C_3_ graminoids in the winter was mostly *Carex* (sedge) species, which often remains green throughout the winter (Fig. 2). C_4_ grass relative read abundance (RRA) averaged 30–40% for most of the year except in early April when it declined to ~10%.

N_2_-fixing species dominated Kansas bison RRA in spring and fall. The seasonal abundance of N_2_-fixing species in the diet was bimodal with RRA peaks of ~70% in late April and ~60% in late September (Fig. 1). The seasonal peak usage was consistent across all three sampling years (Fig. 3). Controlling for day of year and sex of the animal, there were no significant differences among years in C_3_ graminoids, or eudicots (*P* > 0.05). In 2011 compared to 2013 or 2014, C_4_ grasses were a lower proportion of the diet (*P* < 0.001) and N_2_-fixers were a higher proportion of the diet (*P* = 0.001).

Across all dates, 23% of all sequences were from *Ceanothus herbaceus*, an actinorhizal N_2_-fixing shrub. *Ceanothus* abundance in the diet peaked at 60% of the sequences on Apr 24 and 51% on Sep 29. *Ceanothus* was the only plant species that was consumed in different proportions by males and females at either site. It constituted 25% of the sequences in the diet of male Kansas bison, but only 19% for females (*P* < 0.001). *Lespedeza* was the only other N_2_-fixing genus consumed in relatively high proportions, comprising an average of 6% of all sequences. *Lespedeza* abundance in the diet peaked at 26% of total sequences approximately 30 d after the spring peak in *Ceanothus*. Forb RRA peaked near Jul 1 at ~35% as Kansas bison progressively consumed different forb species throughout the year (Fig. 2).

Compared to Kansas bison, South Dakota bison consumed a higher proportion of C_3_ grasses and a lower proportion of N_2_-fixing species, but consumed similar proportions of C_4_ grasses and forbs. Between Apr 4 and Oct 8, 43 ± 4% of the sequences for the South Dakota bison were C_3_ graminoids and 21 ± 3% were C_4_ grasses. For Kansas bison over the same range of dates, grass RRA was half of that of the South Dakota bison (64% vs. 33%, *P* < 0.001). Compared to South Dakota bison, Kansas bison C_3_ grass RRA was lower (4 ± 1%, *P* < 0.001), but C_4_ grass was similar (29 ± 2%, *P* > 0.15). Peak C_4_ grass utilization in the cooler South Dakota site was earlier in the season than at Kansas (Fig. 1) due to differences in the C_4_ grass species relied on most heavily by bison at the different sites. For the South Dakota bison, over 25% of all sequences were from *Bouteloua* species for spring and fall samples. *Bouteloua* species are considered relatively palatable shortgrass species that have an earlier phenology than many Andropogonoid C_4_ grasses. During the summer, when Kansas bison largely were consuming Andropogonoid grasses, only ~5% of the diet of SD bison was from C_4_ grasses (Fig. 2).

The lower consumption of C_3_ grasses in Kansas compared to South Dakota was not offset by greater consumption of forbs (18 ± 2% vs. 23 ± 6% RRA, respectively, *P* > 0.15). Instead, bison at the warmer Kansas site were consuming a greater proportion of N_2_-fixing eudicots than the South Dakota site (50 ± 3% vs. 13 ± 3%, *P* < 0.001). *Lotus* cf. *corniculatus* was the dominant N_2_-fixing eudicot consumed at the South Dakota grassland (~10% of all sequences). *Lotus* consumption peaked in mid-summer rather than during spring and fall (~35% at Jul 1). Shifts in dietary composition by the Kansas bison from C_3_ grasses to N_2_-fixing eudicots is incomplete as the Kansas bison generally experience higher protein and energy stress, gain less weight, and reproduce at lower rate than the South Dakota bison (Supplemental discussion).

The relatively high eudicot RRA of the Kansas and South Dakota bison indicates that bison are not strict grazers. If they were, the large majority of the chloroplast sequences would have been derived from graminoids. For example, zebra (*Equus* sp.) are strict grazers and a similar DNA metabarcoding approach showed that >99% of the chloroplast sequences recovered from zebra feces came from grass^20^. Isotopic analyses of fecal material also supports the importance of eudicots in bison diet, although to a lesser extent than DNA metabarcoding. For the Kansas site, fecal δ^13^C ranged from −24.8 to −16.5‰, corresponding to a range of 35% to 86% C_4_ grass in diet. On average, 60% of bison fecal C was derived from C_4_ grasses. Samples with a higher proportion of C_4_ grass DNA had a higher δ^13^C (r = 0.60, *P* < 0.001, n = 78) indicating a greater proportion of C_4_ plant material in the diet. Metabarcoding consistently quantified lower proportions of C_4_ grass in diet compared to estimates based on isotopic values (29.3% vs. 59.6%, s.e. = 1.4%, *P* < 0.001; Fig. 4). Yet, the two approaches are likely measuring different aspects of diet. Based on physiological first principles, RRA more likely represents the relative intake of protein from a plant species while carbon isotopic analyses relate to relative intake of carbon from different plant species (Supplemental discussion).

The timing of high utilization of N_2_-fixing species coincides with peaks in dietary nutritional quality suggesting that bison seek these plants out for nutritional supplementation. Across the 3 years for which we determined diet composition, the concentration of crude protein ([CP]) in bison diet began increasing in early spring at ~Mar 31 and peaked at May 17 when [CP] was 134 mg CP g^−1^ (Fig. 5). At the date of peak [CP], isotopic analyses of fecal material indicate that only 62% of the C that bison consumed was from C_4_ plants. Yet, the date of peak [CP] occurred when C_4_ grasses comprised only 22% of the RRA whereas N_2_-fixing species comprised 53% of the RRA. The difference in percentages for C_4_ grasses implies that the C_3_ plants bison are consuming have higher protein concentrations than the C_4_ grasses, diluting protein of the C_4_ grasses to lower their RRA relative to the percentage of C from C_4_ plants (Fig. 4). Mid-May protein concentrations of *Andropogon gerardii* leaves are approximately 25% lower compared to *Ceanothus herbaceus* leaves (112.5 vs. 155 mg CP g^−1^, respectively) and decline rapidly as the season progresses^21^,^22^,^23^. Bison are likely selecting for *Ceanothus* during this time. *Ceanothus* is relatively uncommon in the uplands and lowlands at the Kansas grassland (< 1% cover), but averages > 10% of the cover on slope positions. Although eudicot species are more likely to have secondary compounds than grasses, eudicots (especially N_2_-fixing ones) typically have higher protein concentrations than C_3_ or C_4_ grass species (Fig. S1).

Given the important roles of domestic and wild large herbivores in human economies and greenhouse gas production^24^,^25^, more research quantifying seasonal patterns of dietary composition is critical for predicting consequences of climate change to the biosphere. In all, bison and other large herbivores in a warmer world are likely to experience greater nutritional stress^1^,^2^, especially as increasing atmospheric CO_2_ concentrations further reduce plant protein concentrations in grasslands^26^. Increased consumption of N_2_-fixing species by bison with warming may be reducing N inputs into grasslands, which is an important negative feedback to decreasing N availability in grasslands. Though still untested, the principles laid out here also are likely to be important for large domestic herbivores^20^,^27^. Yet, we know little about the actual diet of livestock on native grasslands, no less the wild large herbivores that are also likely facing increasing nutritional pressures as climates warm. If nutritional quality continues to decline, nutritional stress will progressively increase, eventually reducing the size and reproduction of large herbivores and altering their controls on plant biodiversity and ecosystem function. Aerial spraying of herbicides, often used to indiscriminantly increase grass cover at the expense of eudicots to increase the performance of cattle, is not likely to promote future bison performance. From our results here, any plan for bison conservation in a warming world needs to ensure diverse, phenologically balanced communities of critical eudicots, if not directly increasing the protein concentrations of grasses through increased use of prescribed fire or direct fertilization.

## Methods

Fecal collection occurred at two sites. The Kansas bison herd resided at Konza Prairie Biological Station, Kansas, USA (39.10, −96.61). Mean annual temperature is 12.6 °C and mean annual precipitation is 845 mm (1994–2013). At the Kansas site, approximately 400 bison have access to 9.6 km^2^ distributed across 10 watersheds^28^. Within the bison unit, approximately 50% of the area is burned annually with watersheds burned in the spring at different fire frequencies. Vegetation at the Kansas site is dominated by warm-season perennial grasses but many eudicot species are present in grazed areas. Animals graze on natural vegetation available to them and were not supplemented with hay, protein, or energy during the study period.

The South Dakota bison herd resided at the Samuel H. Ordway Memorial Preserve, South Dakota, USA (45.71, −99.10). Mean annual temperature is 6.3 °C and mean annual precipitation is 537 mm (1994–2013). At the South Dakota site, approximately 350 bison have access to approximately 12 km^2^. In contrast to the Kansas site, the bison enclosure at Ordway has not experienced a burn since 2008. Vegetation at the South Dakota site is dominated by perennial cool-season grasses. The bison graze on natural vegetation during the growing season with no supplementation except minerals, although hay occasionally is provided during the winter.

At the Kansas site, fresh fecal material was collected from typically 5 adult females and 5 adult males approximately biweekly during the growing season and monthly from Nov-Mar. For the Kansas bison, samples from 2011, 2013, and 2014 were analyzed here (samples from Nov 2011 through Oct 2012 were lost while in storage). For the South Dakota bison, samples were collected from an average of 3 adult females and 3 adult males approximately monthly from Mar-Oct. At each site, samples were composited by sex and then frozen.

Genomic DNA from fecal and plant samples was extracted using the MoBio PowerSoil htp-96 well Isolation Kit (Carlsbad, CA). A portion of the chloroplast trnL intron was PCR amplified from each genomic DNA sample using the c and h trnL primers^19^, but modified to include appropriate barcodes and adapter sequences for Illumina multiplexed sequencing (Supplementary Methods). Each 25 μL PCR reaction was mixed according to the Promega PCR Master Mix specifications (Madison, WI), with 2 μL of genomic DNA template. Amplicons from each sample were cleaned and normalized using SequalPrep Normalization Plates (Life Technologies, Carlsbad, CA ) prior to being pooled together for sequencing on an Illumina MiSeq (San Diego, CA) running the 2 × 150 bp chemistry. Sequences were demultiplexed, paired end reads were merged, trimmed followed by a quality control step (Supplementary Methods). Sequences were clustered into OTUs at the ≥97% sequence similarity level and sequence abundance counts for each OTU were determined using the usearch7 approach^29^. The National Center for Biotechnology Information (NCBI) genus names associated with each hit were used to populate the OTU taxonomy assignment lists. Upon inspection of experimental blanks, one OTU (representing *Pinus* species) was found to have been a contaminant during the PCR process due to its presence in the negative controls. As *Pinus* species were not present at either site, the data for this OTU were excluded from analyses. On average, we obtained 4630 sequences per sample after exclusion of the *Pinus* OTU. To simplify analyses and presentation of data, only the 50 most abundant OTUs from the KS and SD site are analyzed here, comprising a total of 88% and 94% of all reads, respectively.

To determine the proportion of dietary C derived from C_4_ grasses, carbon isotopic analysis of fecal material were performed on a Thermo Delta V+ isotope ratio mass spectrometer interfaced with a Carlo Erba NC2500 elemental analyzer. The isotopic ratio of ^13^C:^12^C (δ^13^C) is expressed relative to the standard of Pee Dee Belemnite (PDB). To generate the percentage of C_3_ and C_4_ species in bison diet, a dual-mixing model was used (Supplementary Methods). Near infrared spectroscopy of fecal material was used to estimate [CP] based on calibrations between NIRS and directly measured forage quality for cattle (Supplementary Methods). Data on the 2011 cover of *Ceanothus* at Konza Prairie was derived from the PVC02 dataset downloaded from http://konza.ksu.edu on June 26, 2015.

Comparisons of the proportions of diets of different functional groups and species between the sexes were computed with paired t-tests. To examine differences among years in dietary composition at the KS site, a spline (λ = 1000) was fit to the proportion of each of 4 functional groups in the diet. The residuals of each relationship were then tested for differences among years with a least squares regression model that included sex and the identity of the year. Differences among years in the residuals were tested with Tukey’s HSD. Comparison of the proportion of C_4_ plants in the diet as determined by DNA metabarcoding and isotopic values were computed with a reduced major axis analysis and paired t-test. All statistics were computed in JMP v. 11.2 (SAS Institute, Cary, NC, USA) and all graphs produced with R.

## Additional Information

**How to cite this article**: Craine, J. M. *et al.* Climatic warming and the future of bison as grazers. *Sci. Rep.* **5**, 16738; doi: 10.1038/srep16738 (2015).

## Supplementary Material

Supplementary Information

## Figures and Tables

**Figure 1:**
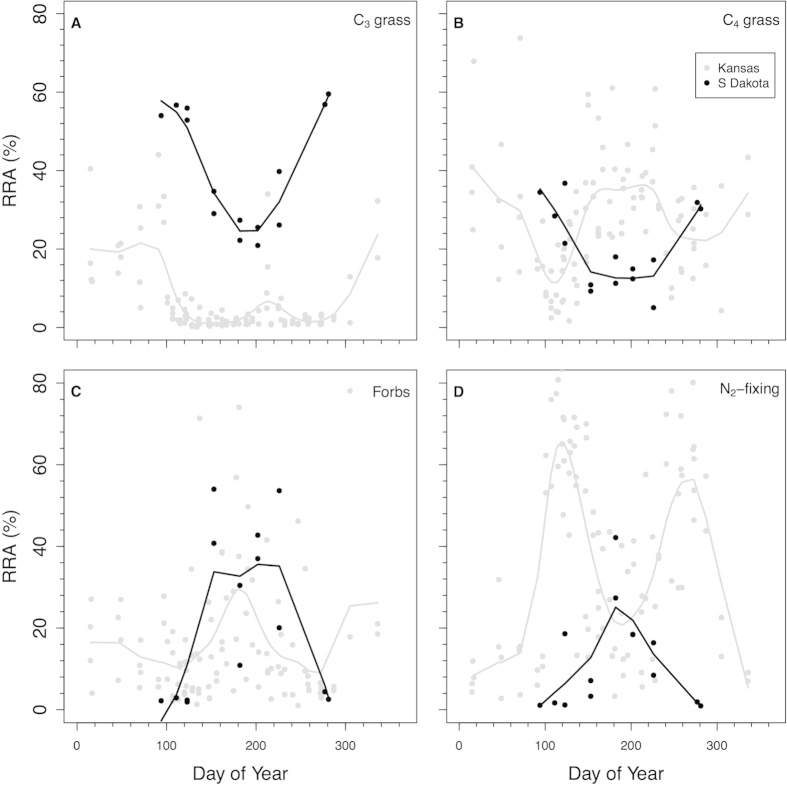
Seasonal pattern of relative read abundances (RRAs) of trnL sequences in bison fecals. (**a**) C_3_ graminoids, (**b**) C_4_ grasses, (**c**) non-N_2_-fixing forbs, and (**d**) N_2_-fixing species for bison in South Dakota (black) and Kansas (grey). Each point is the abundance of sequences pooled across multiple males or females at a given time point. Data shown for 2011, 2013, 2014 for Kansas and 2014 for South Dakota.

**Figure 2:**
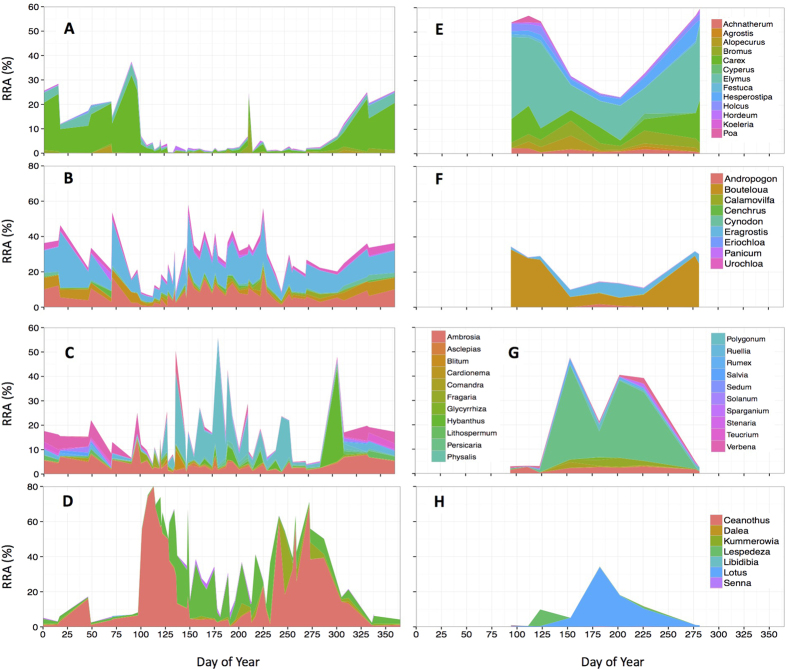
Seasonal pattern of relative read abundances (RRAs) in bison fecals within each of four functional groups. (**a**,**e**) C_3_ graminoids, (**b**,**f**) C_4_ grasses, (**c**,**g**) non-N_2_-fixing eudicots, (**d**,**h**) N_2_-fixing eudicots for Kansas (**a**–**d**) and South Dakota (**e**–**h**) bison. All OTUs were aggregated to shared common genera. RRAs are only shown for the top 50 most abundant OTUs, which comprised an average of 90% of all reads. Data shown for 2011, 2013, 2014 for Kansas and 2014 for South Dakota.

**Figure 3:**
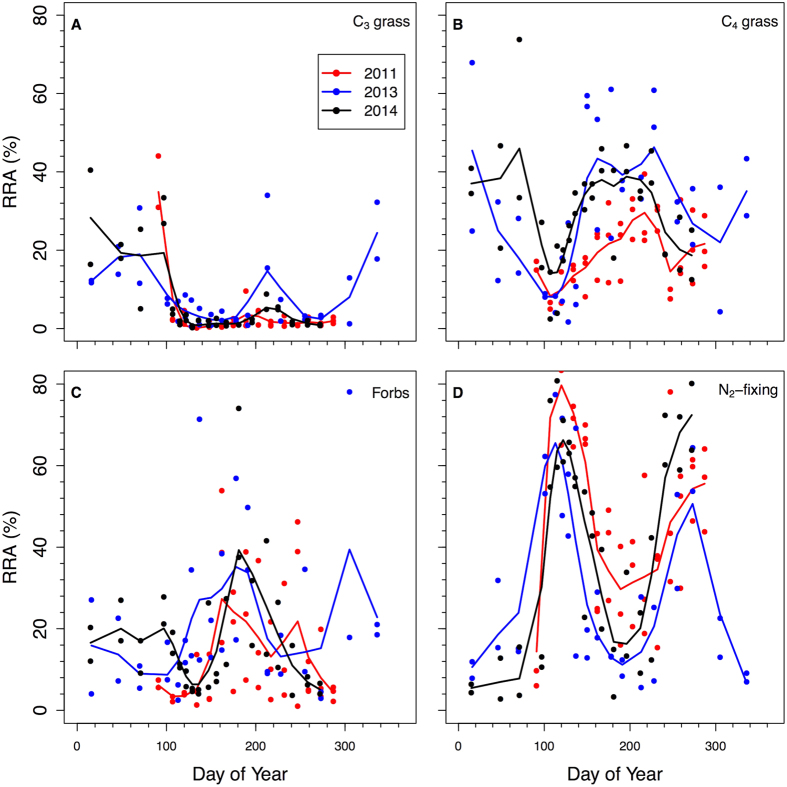
Seasonal pattern of relative read abundances (RRAs) for Kansas bison in 2011, 2013, and 2014. (**a**) C_3_ graminoids, (**b**) C_4_ grasses, (**c**) non-N_2_-fixing forbs, and (**d**) N_2_-fixing species. Each point is the abundance of sequences pooled across multiple males or females at a given time point.

**Figure 4:**
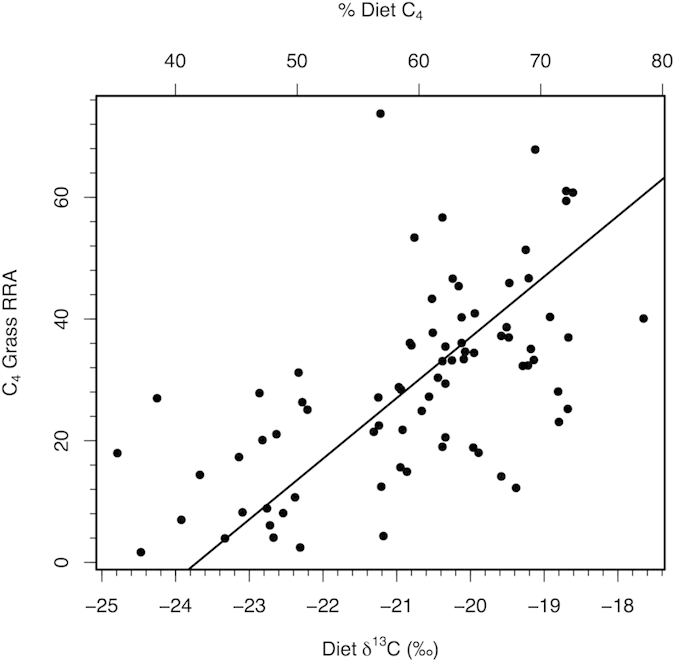
Relationship between the relative amount of C_4_ species in diet as determined by metabarcoding and isotopic analyses of fecals. Isotopic data shown as fecal δ^13^C and % of diet at C_4_ species based on typical values for C_3_ and C_4_ species at the Kansas grassland. Data shown for 2011, 2013, 2014.

**Figure 5:**
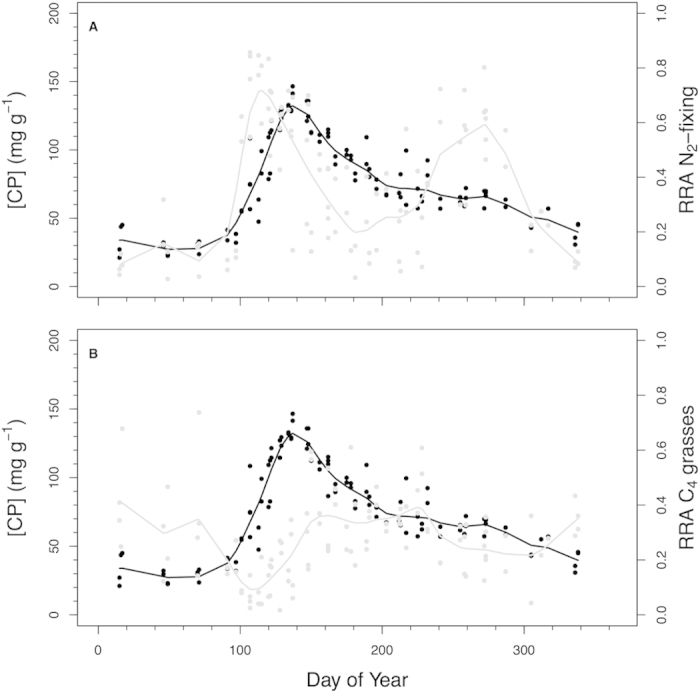
Seasonal patterns of dietary crude protein concentrations ([CP]) for Kansas bison and plant functional group relative read abundance (RRA). Patterns of [CP] shown vs. RRAs for (**a**) N_2_-fixing plants and (**b**) C_4_ grass. Data shown for 2011, 2013, 2014. Black points and line for South Dakota bison; gray points and line for Kansas bison.
