# Supplementary material for: Climatic warming and the future of bison as grazers
Source: Sci Rep. 2015 Nov 16;5:16738. doi: 10.1038/srep16738 (PMC4645125; doi:10.1038/srep16738)
Supplement: Supplementary Information [file srep16738-s1.pdf]

Supplement for:

Climatic warming and the future of bison as grazers

Joseph M. Craine, E. Gene Towne, Mary Miller, Noah Fierer

Supplemental methods

The barcodes used were 12-bp error-correcting barcodes unique to each sample[1]. The theromocycling program used an initial step at 94 °C for 2 minutes, a final extension at 72 °C for 2 minutes and the following steps cycled 35 times: 2 minutes at 94 °C, 1 minute at 55 °C, and 30 seconds at 72 °C. Sequences were demulitplexed using a python script available from: https://github.com/leffj/helper-code-for-uparse/blob/master/prep_fastq_for_uparse_paired.py Paired end reads were then merged using fastq_merge pairs[2]. Since merged reads often extended beyond the amplicon region of the sequencing construct, we used fastx_clipper to trim primer and adaptor regions from both ends (https://github.com/agordon/fastx_toolkit). Sequences lacking a primer region on both ends of the merged reads were discarded.

Sequences were quality trimmed to have a maximum expected number of errors per read of less than 0.1 and only sequences with more than 3 identical replicates were included in downstream analyses. BLASTN 2.2.30+ was run locally, with a representative sequence for each OTU as the query and the current NCBI nt nucleotide and taxonomy database as the reference. The tabular BLAST hit tables for each OTU representative were then parsed so only hits with > 97% query coverage and identity were kept.

In the isotopic mixing model, the diet-feces carbon isotopic offset was set to -0.9‰[3,4]. Reference C_3 and C_4 $\delta^{13}C$ values were derived from a survey of herbaceous Konza plant species collected at the time of first flowering[5,6]. These included 309 herbaceous C_3 species (-29.7 ± 0.08 ‰) and 56 herbaceous C_4 species (-13.2 ± 0.2 ‰). Data on foliar N

concentrations (Fig. S1 and *Ceanothus herbaceus* in text) were taken from ref 6. A conversion of 6.25:1 was used between [CP] and [N][7].

For NIRS analysis, after collection, fecal samples were frozen and later dried at 60 $^{\circ}$C in a forced air oven, ground to 1 mm particle size and re-dried at 60 $^{\circ}$C prior to scanning with near infrared spectroscopy (NIRS) by Texas A&M's Grazingland Animal Nutrition Lab[8,9]. Spectra (400–2500 nm) were collected on a Foss NIRS 6500 scanning monochrometer with spinning cup attachment. Reference chemistry and chemometrics for NIRS calibration development that link forage chemistry and fecal spectra were generated for cattle[10].

Supplemental discussion

OTU Richness

The richness of OTUs in diet for bison in the Kansas and South Dakota grasslands are similar. In the Kansas grassland, an average of 14.1 ± 0.5% distinct OTUs per bison fecal sample were recovered with a total of 65 OTUs detected across all samples per year (minimum 1% relative read abundance). At the South Dakota grassland, bison consumed a similar richness of species in their diet as in Kansas—an average of 13.8 OTUs per sample from DOY 94-281 vs. 14.0 OTUs for the same period ($P > 0.85$). During this time period, as the season progressed, dietary richness increased (DR = 9.49 + 0.025*DOY, $r^2 = 0.09$, $P = 0.0026$) with no significant difference between sexes or sites in the relationship ($P > 0.05$).

Biomass vs. protein intake

The *trnL* sequence examined here is a chloroplast intron with no known variation in copy number[11]. Because chloroplast density scales with foliar nitrogen concentrations and therefore protein concentrations [7,12], the percentage of trnL sequences in fecal material should scale with the percentage of protein ingested from that plant species. That metabarcoding provides information on protein rather than C or biomass intake is not necessarily a drawback given that many herbivores are more limited by protein than energy[13], allowing for resolution of relative importance of different plant species for a limiting nutrient to herbivores.

A feeding trial has indicated a strong 1:1 relationship between biomass intake and RRA[14], but this could be explained by the two plant species used in the feeding trial having similar protein concentrations. The relatively greater proportion of C_4 species in bison diet here suggested by isotopic analysis as opposed to metabarcoding likely reflects the lower N concentrations of C_4 grasses relative to other species (Fig. S1). The slope of the relationship between the proportion of C_4 plants in bison diet estimated with isotopes and C_4 grass RRA indicates that the underrepresentation was greatest when C_4 grass component of diet was lowest (Fig. 4), which is probably the time of the greatest difference in protein concentrations between C_4 grasses and eudicots.

Forage quality differences between sites

Forage quality is primarily defined by the concentrations of crude protein (CP) and digestible organic matter (DOM) in plant matter consumed by grazers [7]. When the ratio of concentrations of DOM to CP is relatively high (e.g. > 4), weight gain is determined more by the concentration of CP in forage than DOM[15]. Typically, CP is more limiting to weight gain in North American grazers. In a synthesis of continental-scale patterns of forage quality, dietary DOM:CP for cattle on pastures and rangelands was generally high indicating greater protein-limitation than energy-limitation [13]. Geographic patterns in forage quality characteristics also demonstrated that protein limitation became greater as MAT increased as peak spring CP concentrations decreased with increasing temperatures[13].

In 2003, we had measured seasonal patterns of [CP] at the South Dakota grassland and found that [CP] had peaked at 182 mg g^{-1} in May. This is 48 mg g^{-1} greater than peak [CP] for the Kansas grassland (ref 16 and this study). Concomitant with the greater measured forage quality, bison weights were also higher in the South Dakota than the Kansas grassland. For example, bison calf weight is a good index of the forage quality available to bison in a particular year. From 2004-2008—the first 5 years weights were measured at the South Dakota grassland—calf weights averaged 199 kg (Fig. S2). By comparison, the long-term average calf weight at the Kansas grassland was 134 kg [16]. Reproduction rates for adult females were also higher at the South Dakota than the Kansas grassland (87% vs. 65%, respectively).

Recently, it appears that forage quality has declined at the South Dakota site. When forage quality was measured on the 2014 South Dakota fecals, the highest [CP] measured was only 100 mg g^{-1} (Fig. S3). Part of the relatively low peak [CP] might be influenced by the relatively low frequency of sampling which could have missed peak forage quality. Yet, 2014 South Dakota [CP] was lower throughout the year than in 2003 and were now similar to the Kansas grassland. [DOM] concentrations were also lower than in 2003, but still higher than the for the Kansas grassland. Examining the weights of bison from 2010-2013 in the SD grassland (bison weights were not measured in 2009 or 2014), bison calf weights were lower than 2004-2008, too, declining by an average of 26 kg (e.g. calf weights were 199.8 vs. 173.2 kg, 2010-2013 vs. 2004-2008 respectively; $P = 0.005$), though still higher than the long-term average calf weights for the KS grassland.

Comparing the two periods, there was little difference in monthly precipitation patterns or mean temperatures that could explain the decline in forage quality and bison weights in the SD grassland over the ten-year period. However, budget reductions had forced a cessation of prescribed fires in 2007. No prescribed fires have been set since then. Late-spring prescribed fires had been implemented in 2004, 2005, and 2006 and there was a unprescribed fire in the spring of 2008. In these 4 years, an average of approximately 140 ha was burned each year, which is approximately 10% of the area available to the bison. Spring fires increase the protein concentrations of grass available to grazers by removing senesced material and increasing the protein concentrations of the new regrowth[17,18].

In all it appears that when fire was removed from the South Dakota grassland, forage quality and weight gain declined. Cool-season grasses such as *Poa pratensis* and *Bromus inermis* have also increased in abundance (M.M., personal observation), likely in

response to the removal of May fires, but these species appear to be little utilized by bison. Although bison weight gain and reproduction is still greater at the South Dakota than the Kansas grassland, bison could not compensate for the decline in grass quality that accompanied the cessation of burning by increasing their consumption of high-protein forbs. Although there are still questions regarding the diet of bison on climatically cool grasslands that have recently experienced fire, the decline in bison performance with cessation of fire further evince the importance of forage quality and landscape-level community composition in limiting the resilience of herbivores to changes in grasslands that are likely to accompany climatic warming.

1 Caporaso, J. G. *et al.* Ultra-high-throughput microbial community analysis on the Illumina HiSeq and MiSeq platforms. *The ISME journal* **6**, 1621-1624, (2012).

2 Edgar, R. C. Search and clustering orders of magnitude faster than BLAST. *Bioinformatics* **26**, 2460-2461, (2010).

3 Sponheimer, M. *et al.* An experimental study of carbon-isotope fractionation between diet, hair, and feces of mammalian herbivores. *Can. J. Zool.* **81**, 871-876, (2003).

4 Codron, D., Codron, J., Lee-Thorp, J. A., Sponheimer, M. & de Ruiter, D. Animal diets in the Waterberg based on stable isotopic composition of faeces. *S. Afr. J. Wildl. Res.* **35**, 43-52, (2005).

5 Craine, J. M., Towne, E. G., Ocheltree, T. W. & Nippert, J. B. Community traitscape of foliar nitrogen isotopes reveals N availability patterns in a tallgrass prairie. *Plant Soil* **356**, 395-403, (2012).

6 Craine, J. M., Wolkovich, E. M., Towne, E. G. & Kembel, S. W. Flowering phenology as a functional trait in a tallgrass prairie. *New Phytol.* **193**, 673-682, (2012).

7 Van Soest, P. J. *Nutritional Ecology of the Ruminant*. 2nd edn, (O & B Books, 1994).

8 Lyons, R. K. & Stuth, J. W. Fecal NIRS equations for predicting diet quality of free-ranging cattle. *J. Range Manag.* **45**, 238-244, (1992).

9 Roberts, C. A., Stuth, J. & Flinn, P. in *Near Infrared Spectroscopy in Agriculture* (eds C A Roberts, J Workman, & J B Reeves) 231-268 (American Society of Agronomy-Crop Science Society of America-Soil Science Society of America, 2004).

10 Showers, S. E., Tolleson, D. R., Stuth, J. W., Kroll, J. C. & Koerth, B. H. Predicting diet quality of white-tailed deer via NIRS fecal profiling. *Rangeland Ecology and Management* **59**, 300-307, (2006).

11 Taberlet, P., Gielly, L., Pautou, G. & Bouvet, J. Universal primers for amplification of three non-coding regions of chloroplast DNA. *Plant Mol. Biol.* **17**, 1105-1109, (1991).

12 Evans, J. R. & Seeman, J. R. in *Photosynthesis* (ed W R Briggs) 183-205 (Liss, 1989).

13 Craine, J. M., Elmore, A. J., Olson, K. C. & Tolleson, D. Climate change and nutritional stress in cattle. *Global Change Biology* **16**, 2901-2911, (2010).

14 Willerslev, E. *et al.* Fifty thousand years of Arctic vegetation and megafaunal diet. *Nature* **506**, 47-51, (2014).

15 Moore, J. E., Brant, M. H., Kunkle, W. E. & Hopkins, D. I. Effects of supplementation on voluntary forage intake, diet digestibility, and animal performance. *J. Anim. Sci.* **77**, 122-135, (1999).

16 Craine, J. M., Towne, E. G., Tolleson, D. & Nippert, J. B. Precipitation timing and grazer performance in a tallgrass prairie. *Oikos* **122**, 191-198, (2013).

17 Allred, B. W., Fuhlendorf, S. D., Engle, D. M. & Elmore, R. D. Ungulate preference for burned patches reveals strength of fire-grazing interaction. *Ecol Evol* **1**, 132-144, (2011).

18 Van de Vijver, C., Poot, P. & Prins, H. H. T. Causes of increased nutrient concentrations in post-fire regrowth in an East African savanna. *Plant Soil* **214**, 173-185, (1999).

Figure S1| Frequency among plant species of foliar N concentrations ([N]) in Kansas grassland by functional group. Functional groups include C_3 grass [C3G], C_4 grass [C4G], non-N_2-fixing herbaceous eudicots [Forb], and N_2-fixing eudicots [N_2-fix]. Foliar [N] for each species were measured on first day of flowering.

Figure S2| Fall weights of male and female bison calves over time at South Dakota Prairie.

Figure S3| Crude protein (CP) and digestible organic matter (DOM) concentrations for the Kansas grassland (2011, 2012, 2013) and the South Dakota grassland (2003, 2014).

Figure S1.

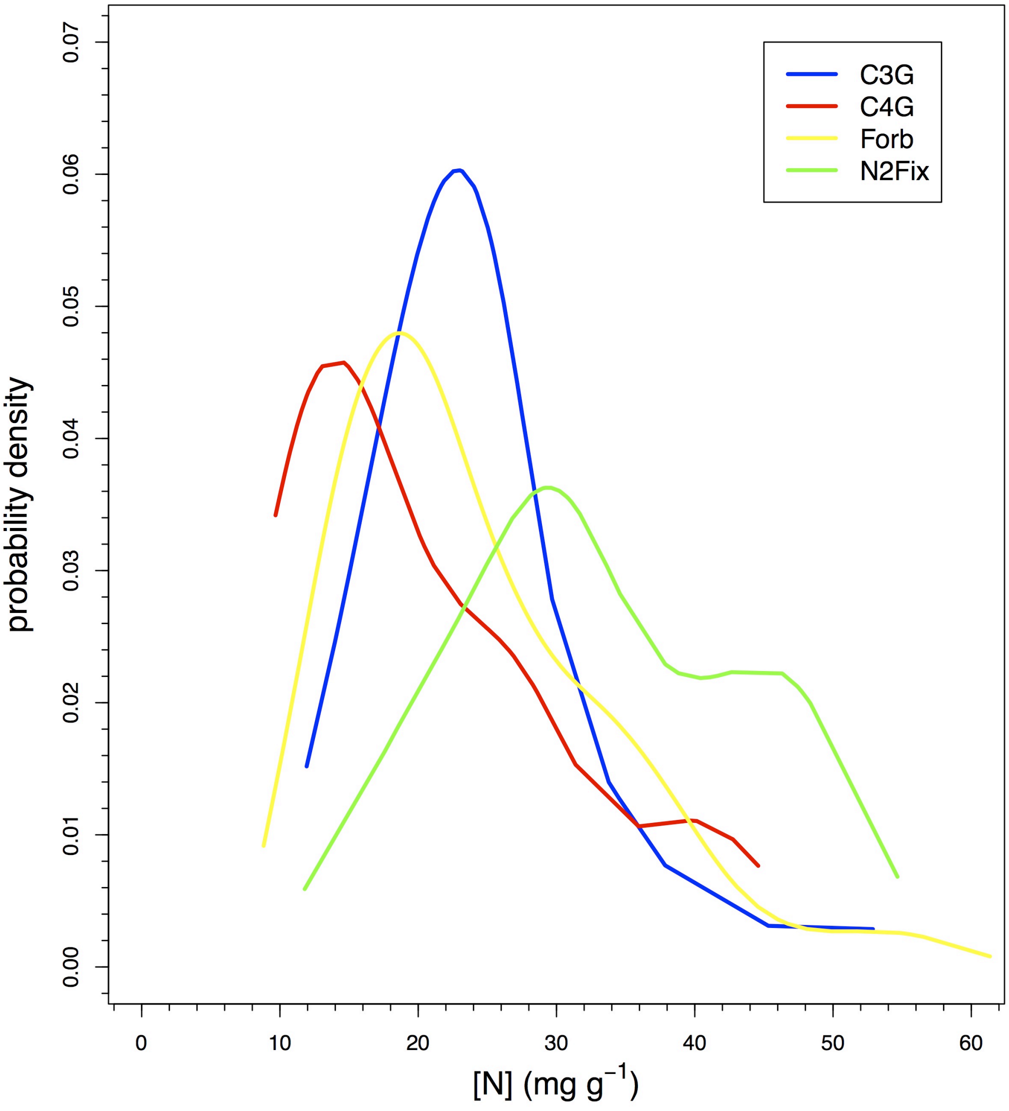

C3G
C4G
Forb
N2Fix
probability density
0.00
0.01
0.02
0.03
0.04
0.05
0.06
0.07
0
10
20
30
40
50
60
[N] (mg g^{-1})

Figure S2.

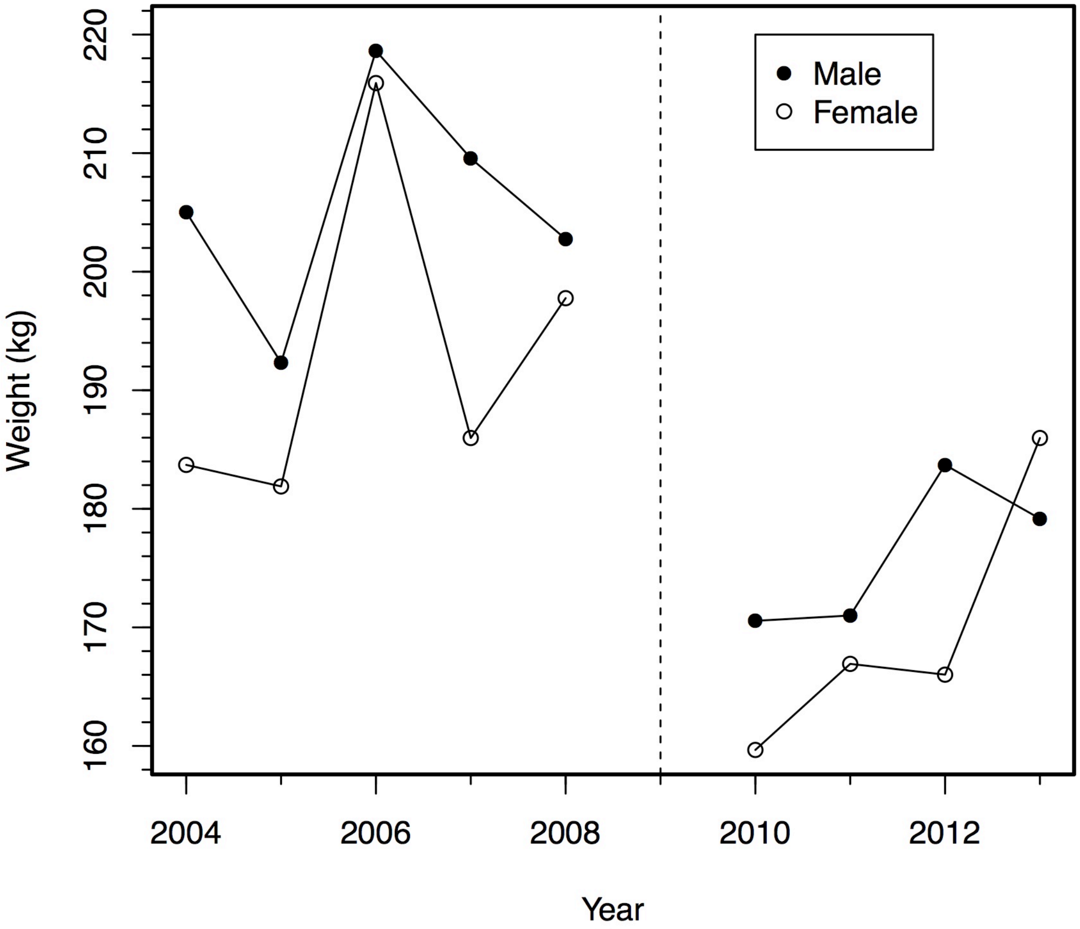
Male
Female
Weight (kg)
Year
160
170
180
190
200
210
220
2004
2006
2008
2010
2012

Figure S3.

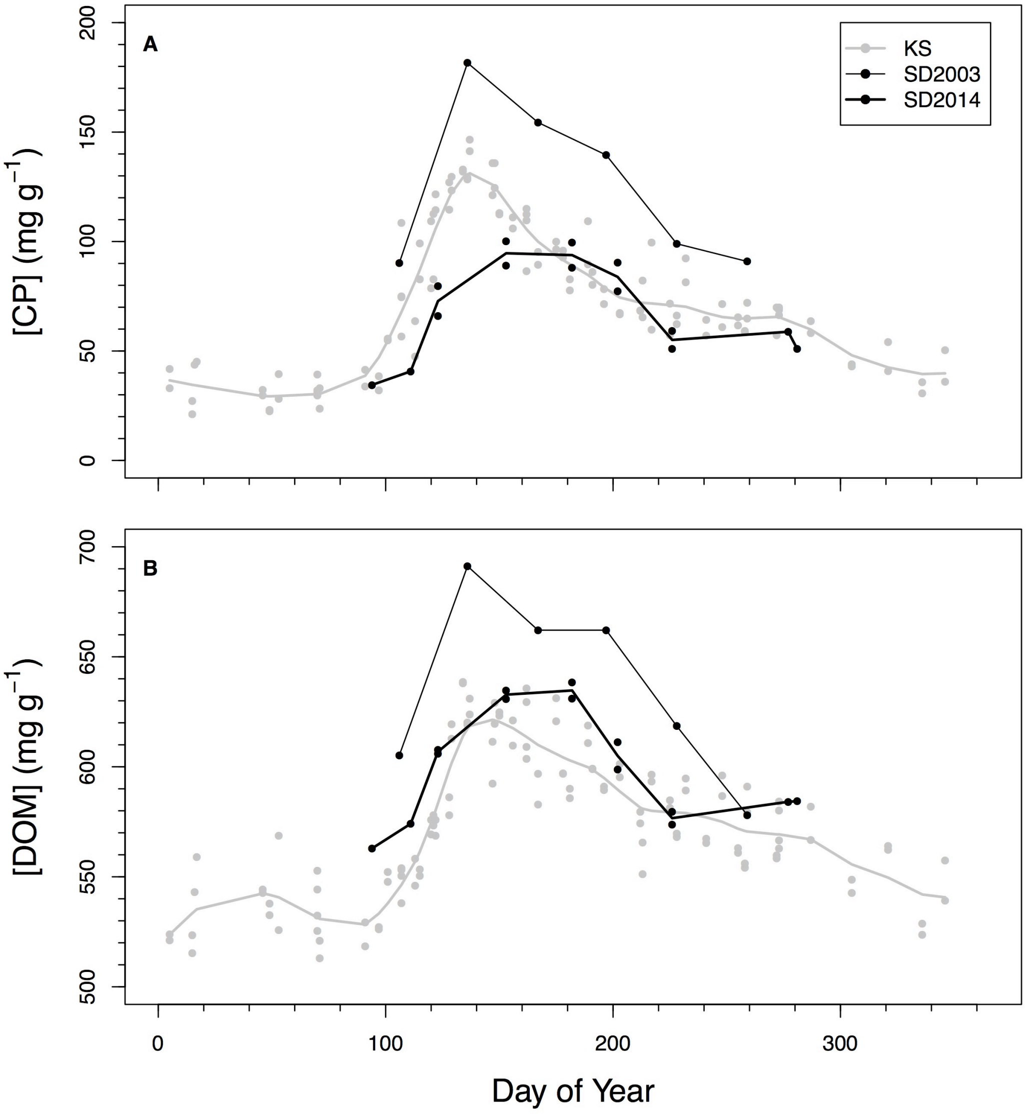
A
B
KS
SD2003
SD2014
[CP] (mg g^{-1})
[DOM] (mg g^{-1})
Day of Year
0
50
100
150
200
500
550
600
650
700
0
100
200
300
